# Functional Role of Monocytes and Macrophages for the Inflammatory Response in Acute Liver Injury

**DOI:** 10.3389/fphys.2012.00056

**Published:** 2012-10-19

**Authors:** Henning W. Zimmermann, Christian Trautwein, Frank Tacke

**Affiliations:** ^1^Department of Medicine III, RWTH-University Hospital AachenAachen, Germany

**Keywords:** liver injury, acute liver failure, macrophages, monocytes, TNF-alpha, chemokines, CCR2, review

## Abstract

Different etiologies such as drug toxicity, acute viral hepatitis B, or acetaminophen poisoning can cause acute liver injury or even acute liver failure (ALF). Excessive cell death of hepatocytes in the liver is known to result in a strong hepatic inflammation. Experimental murine models of liver injury highlighted the importance of hepatic macrophages, so-called Kupffer cells, for initiating and driving this inflammatory response by releasing proinflammatory cytokines and chemokines including tumor necrosis factor (TNF), interleukin-6 (IL-6), IL-1beta, or monocyte-chemoattractant protein-1 (MCP-1, CCL2) as well as activating other non-parenchymal liver cells, e.g., endothelial or hepatic stellate cells. Many of these proinflammatory mediators can trigger hepatocytic cell death pathways, e.g., via caspase activation, but also activate protective signaling pathways, e.g., via nuclear factor kappa B (NF-κB). Recent studies in mice demonstrated that these macrophage actions largely depend on the recruitment of monocytes into the liver, namely of the inflammatory Ly6c+ (Gr1+) monocyte subset as precursors of tissue macrophages. The chemokine receptor CCR2 and its ligand MCP-1/CCL2 promote monocyte subset infiltration upon liver injury. In contrast, the chemokine receptor CX3CR1 and its ligand fractalkine (CX3CL1) are important negative regulators of monocyte infiltration by controlling their survival and differentiation into functionally diverse macrophage subsets upon injury. The recently identified cellular and molecular pathways for monocyte subset recruitment, macrophage differentiation, and interactions with other hepatic cell types in the injured liver may therefore represent interesting novel targets for future therapeutic approaches in ALF.

## Introduction

Acute liver injury (ALI) and acute liver failure (ALF) represent different severity stages of a sudden deterioration of liver function without evidence for prior chronic liver disease. It is a dreaded disease condition due to its tremendous morbidity and mortality without adequate treatment. Clinical hallmarks of ALF are coagulopathy (defined as an INR > 1.5) and mental alterations (i.e., hepatic encephalopathy of any degree) within a 26-weeks time-frame after the initial symptoms (Bernal et al., 2010). The latter clinical condition is absent in ALI in which coagulation abnormalities are predominant. The annual incidence of ALF is estimated at one to six cases per million in the developed world but may be higher in endemic regions of viral hepatitis (Bernal et al., 2010). Due to insufficient surveillance and reporting systems and lack of consistent diagnostic criteria accurate data concerning the global epidemiology of ALI are scarce. Drug-induced liver injury as a common underlying cause is estimated to affect 44,000 individuals in the US per year (Bell and Chalasani, 2009). Miscellaneous causes of acute liver deterioration exist, and etiology is the best predictor of clinical outcome (Ostapowicz et al., 2002). In major parts of the western hemisphere acute dose-dependent acetaminophen (paracetamol) toxicity is the most prevalent cause of ALF, whereas viral agents (mainly hepatitis A, B, or E virus) predominate in developing countries. In recent years, idiosyncratic, non-acetaminophen, drug-induced hepatotoxicity became a major etiology of ALF in Europe (Canbay et al., 2011). ALF is a systemic disease. Owing to its devastating nature implications of liver failure rapidly affect virtually all vital organs eventually leading to multi-organ failure. Despite remarkable progress in disease management and understanding of basic molecular mechanisms involved, disease-specific, targeted therapies cannot be provided in a considerable proportion of cases where liver transplantation constitutes the sole medical mean to prevent death.

Local and circulatory components of the innate immune system fundamentally shape the outcome of the immunological response to an acute hepatic insult. There is a robust body of evidence that hepatic macrophages (traditionally called “Kupffer cells,” KCs) are essential players in the propagation of acute liver damage. These cells attracted much attention lately in the context of chronic liver inflammation due to their dual pro- and antifibrotic qualities (Zimmermann and Tacke, 2011) but evidence for their critical involvement in fulminant hepatitis even date back several decades. Ever since, the evolvement of intriguing techniques to impact KC function has paved the way for a deepened knowledge and enabled us to decipher detrimental as well as beneficial aspects of KC activity. The present review intends to focus on hepatic macrophages in ALI as well as on monocytes, the bone-marrow-derived macrophage precursors that are vigorously recruited upon liver damage. Chemokine pathways governing this process will be a main focus in the subsequent sections, because interference with these pathways might perspectively allow developing novel and effective therapeutic approaches for ALF in the near future.

## Resident and Infiltrating Hepatic Macrophages during Homeostasis and Injury

### General aspects of liver anatomy and microvasculature

The liver is not only the largest solid organ of the human body but also possesses the most extensive reticuloendothelial system (RES), thus playing a central role in the immune response against invading pathogens. It is unique in its property as an organ that encounters all the foreign material adsorbed from the intestine after digestion including food-derived antigens and environmental toxins (Gao et al., 2008). In addition, blood floating into the liver via the portal vein (accounting for ∼80% of total liver blood supply) contains microbial components even under steady state conditions with lipopolysaccharide (LPS) from gut-derived Gram-negative germs representing some of the most prominent bacterial constituents. Moreover, due to an arterial blood supply the liver also samples antigens from systemic circulation. The hepatic microvasculature is composed of liver sinusoids that are lined by highly specialized liver sinusoidal endothelial cells (LSEC) that tremendously differ from generic vascular endothelium in terms of phenotype, surface markers, and function (Lalor et al., 2006). Portal venous and arterial vessel branches supply the sinusoids with blood. Sinusoidal fenestrations and the lack of a basal membrane facilitate the delivery of solutes across the subendothelial space of Dissé to the hepatocytes which constitute the hepatic parenchyma. Signals evoked by invading macro-material and cellular effectors rely on active recruitment via sinusoidal cells or endocytosis/phagocytosis and cytokine-release of resident phagocytic cells. Following drainage into the central vein the “liver-modified” blood reaches systemic circulation through the vena cava inferior.

### Kupffer cells are resident macrophages and fulfill essential tasks during steady state

**Figure 1:**
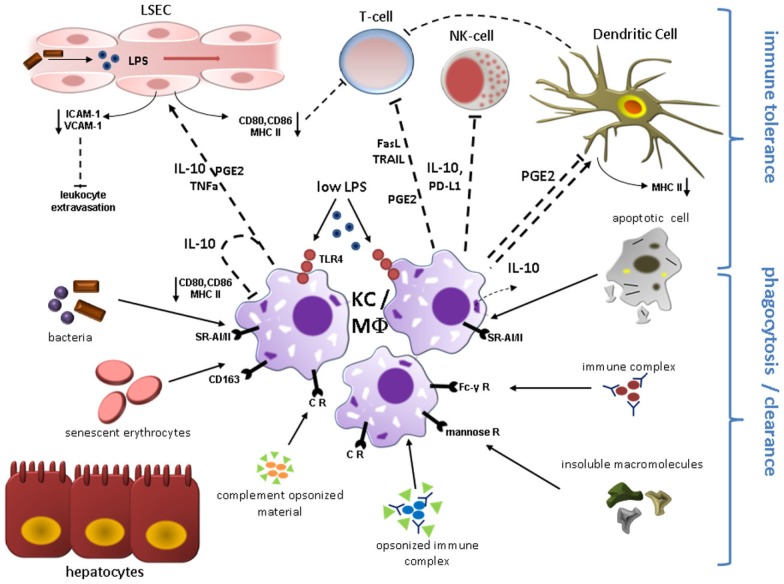
**Kupffer cell (KC)/Macrophage (MΦ) function during liver homeostasis**. Phagocytosis and induction of immune tolerance as the two main functions of KCs/hepatic MΦ in the steady state are depicted here. KCs reside in the liver sinusoids in close proximity to sinusoidal endothelial cells (LSEC) and immune cells entering the liver microvasculature mainly through the portal vein. KCs express a broad range of surface receptors mediating phagocytosis, which renders these cells as highly effective filters of endogenous and exogenous antigens. Complement receptors mediate removal of complement-opsonized material. Circulating non-opsonized immune globulin complexes are cleared through Fc-γ Receptors. Insoluble macromolecules from multiple sources are effectively cleared after binding to Scavenger Receptors including CD163 for senescent erythrocytes. Molecules with a mannosyl motif are phagocytized following engagement of mannose receptors. Engulfment of apoptotic cell constituents can induce secretion of immunosuppressive IL-10 which likely contributes to the immune modulatory function of quiescent KCs. Constant exposure to gut-derived LPS via TLR4 also results in expression of IL-10 and PGE2 that can directly inhibit T-cell and NK-cell function and mediate down-regulation of co-stimulatory proteins including CD80, CD86, and MHC class II on endothelial cells, dendritic cells, and KCs constituting liver APCs, which further attenuates T-cell activation. KC-secreted PD-L1 and release of apoptosis-inducing mediators (TRAIL, FasL) contribute to suppression of adaptive and innate immune response through inactivation/elimination of T-cells and NK-cells. IL-10, PGE2, and TNF-alpha lead to reduced expression of adhesion molecules (VCAM-1; ICAM-1) on LSEC, thereby limiting leukocyte influx. Abbreviations: APC, antigen presenting cell; CR, complement receptor; FasL, Fas ligand; ICAM-1, intercellular adhesion molecule 1; PD-L, programmed cell death 1 ligand 1; PGE2, prostaglandin E2; SR-AI/II, scavenger receptor AI/AII; TLR4, toll-like receptor 4; TRAIL, tumor necrosis factor related apoptosis-inducing ligand; VCAM-1, vascular adhesion molecular 1.

Owing to the direct vascular connection to the splanchnic organs as a source of potential environmental and inherent threats, integral parts of the innate immune system are highly enriched in the liver. This renders the liver as an immunological organ with predominant innate immune functions (Racanelli and Rehermann, 2006; Gao et al., 2008). Apart from resident immune cells that respond to exterior and interior damaging influences, the liver is also source of a host of soluble factors encompassing acute-phase-proteins, complement factors, cytokines, and chemokines, which all contribute to the meticulous orchestration of immune response to various stimuli (Ishibashi et al., 2009). However, the liver is perpetually confronted with harmless nutrient-borne antigens and low levels of LPS and other microbial products. Those do not represent an inflammatory stimulus in steady state conditions but elicit immunosuppressive responses in order to prevent constant detrimental immune activation (Tacke et al., 2009). Intrahepatic macrophages accommodate for both opposing scenarios: promoting immune tolerance during homeostasis as well as implementing proinflammatory mechanisms in acute and chronic liver injury. KCs traditionally denote hepatic (resident) macrophages and represent up to 80–90% of the total body macrophage pool (Ishibashi et al., 2009). Together with LSEC, hepatic stellate cells (HSC), and local immune cells [in particular atypical T-cells, NK-cells (pit cells), and hepatic dendritic cells (DCs)] KCs constitute the non-parenchymal liver cells. They dwell in the lumen of liver sinusoids in close contact to the sinusoidal endothelial cells and sense the circulating blood for food-borne antigens and microbial constituents stemming from the splanchnic circulation. The sinusoidal site also guarantees intimate contact and communication with immune cells that enter the liver via the portal vein. In the non-inflamed liver one of the key functions of KCs is the removal of insoluble macromolecules through phagocytosis mediated by a wide repertoire of pattern-recognition receptors (PRRs) on their surface including scavenger receptors SR-AI and SR-AII, mannose receptor, and Fc-γ receptors (Gao et al., 2008; Figure 1). Thereby, hepatic macrophages eliminate potential harmful threats elicited by degenerated cells, microbes, immune complexes, and toxins (Kolios et al., 2006). KC show functional disparities related to their localization within the liver lobule (Bilzer et al., 2006). Periportal KCs, which are the first macrophages to encounter inflowing portal blood, are more abundant, bigger in size, and exhibit greater phagocytic and lysosomal capacities in addition to an increased production of inflammatory mediators [such as interleukin-1 (IL-1) and tumor necrosis factor (TNF) alpha (TNF-alpha)], in comparison to those hepatic macrophages located in the midzonal area and around the central vein (Sleyster and Knook, 1982; Hoedemakers et al., 1995). Moreover, KCs are not entirely static in the sinusoidal lumen but migrate across the sinusoidal walls and are capable of reducing the sinusoidal blood velocity hence supporting the contact of circulating immune cells with sinusoidal endothelial cells (MacPhee et al., 1992, 1995).

### Origin and phenotype of KCs during absence or presence of hepatic injury

**Figure 2:**
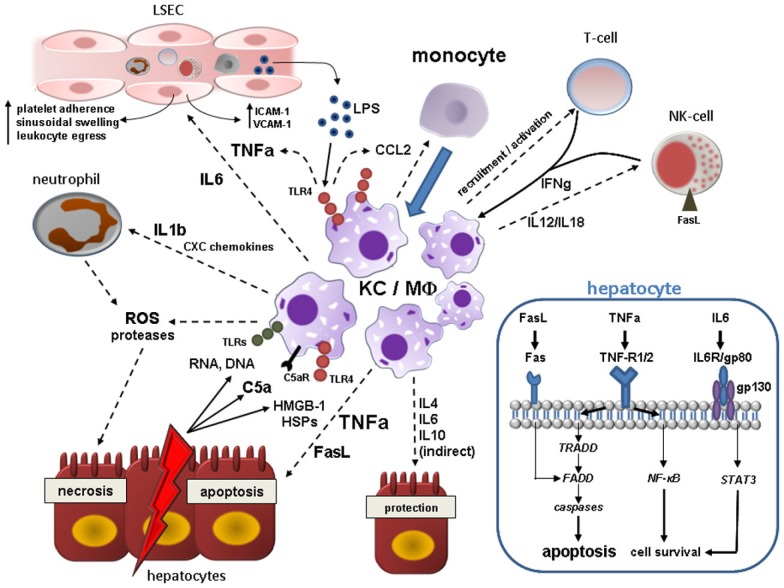
**Kupffer cell (KC)/Macrophage (MΦ) contribution to acute liver injury**. Acute hepatocyte damage in response to multitude events leads to release of various DAMPs including HSPs and HMGB-1, which bind to TLR4 on KCs, and other cell contents (RNA, DNA) binding to various TLRs. TLR4 engagement activates NF-κB pathways in KC resulting in the synthesis of a myriad of proinflammatory cytokines, chemokines, reactive oxygen, and nitrogen species. KC-secreted TNF-alpha is central in the augmentation of liver injury mainly by inducing hepatocyte apoptosis, but also by deterioration of hepatic microcirculation through swelling and activation of endothelial cells with subsequent sinusoidal platelet aggregation and facilitation of peripheral immune cells entry. Activated KCs secrete IL-1beta and CXC chemokines such as CXCL2 and CXCL8 (IL-8), whereby neutrophils are massively attracted and start releasing ROS and proteases evoking hepatocyte necrosis. In addition, KCs, injured hepatocytes and activated hepatic stellate cells secrete CCL2 and other CC chemokines mediating liver influx of bone-marrow-derived monocytes that expand the local macrophage pool. Hepatic macrophages are also stimulated by IFN-gamma from resident and recruited T-cells and NK-cells and by C5a. KCs actively govern NK cell activation and recruitment by the production of IL-12/IL-18 which in turn induces hepatocyte death via membrane-bound FasL. High levels of LPS arising from Gram-negative bacteria in the context of increased bacterial translocation during acute liver injury magnify the activation the liver macrophages. As a counter-regulatory effect, hepatic macrophages also secrete IL-4, IL-6, and IL-10 amongst others that may dampen hepatic injury by either direct or indirect mechanisms. Engulfment of cell debris may either contain or amplify tissue injury (not depicted here). The blue box in the right lower corner illustrates important proapoptotic and prosurvival pathways in hepatocytes after binding of KC-related effector molecules during acute liver injury. TNF-alpha elicits both apoptosis (mainly via TNF-R1 and subsequent downstream signaling involving TRADD, FADD, and effector caspases) and cell survival through activation of NF-κB downstream cascade. Soluble and membrane-bound FasL derived from activated KCs and NK-cells also leads to caspase-dependent cell death after direct engagement of FADD. IL-6 complexes with gp80 and gp130 resulting in STAT3 activation via Janus kinases (JAKs) that can promote cell protection. Abbreviations: C5a, complement factor 5a; FADD, Fas-associated death domain; HMGB-1, high mobility group box-1; HSP, heat-shock protein; IL, interleukin; NF-κB, nuclear-factor-kappaB; ROS, reactive oxygen species; STAT3, signal transducer and activator of transcription 3; TNFa, tumor necrosis factor alpha; TRADD, TNF receptor associated death domain.

In absence of liver inflammation, the number of intrahepatic macrophages is maintained at constant numbers. Various cytokines comprising IL-1, IL-4, interferon-gamma (IFN-gamma), granulocyte–macrophage colony-stimulating factor (GM-CSF), and other hematopoietic factors promote macrophage apoptosis and survival *in vitro* (Naito et al., 2004). Results of studies covering the life span of KCs are inconsistent and range between 14 days and several months (Naito et al., 2004). Interestingly, even in monocytopenic species KC persistence *in situ* may exceed 6 weeks, suggesting that KCs constitute long-lived resident macrophages (Naito et al., 2004). Nevertheless, constant turnover is present and hepatic macrophages are incessantly repopulated. Previous concepts of resident macrophages deriving from precursor cells (Yamamoto et al., 1996; Naito et al., 1997; Duffield et al., 2005) have been refuted by more recent studies that indicate that a significant extent of repopulation of these cells is from bone-marrow-derived myeloid precursors (Klein et al., 2007). In line, in one study only 1.5% of hepatic macrophages incorporated ^3^H-thymidine during steady state, indicative of a low proliferation index (Crofton et al., 1978). In acute and chronic liver injury the intrahepatic macrophage count is massively expanded following the influx of peripheral monocytes (Figure 2) rather than augmentation of tissue-resident macrophages (Duffield et al., 2005; Imamura et al., 2005; Holt et al., 2008; Karlmark et al., 2009; Zimmermann et al., 2010). However, a current paper indicating that IL-4 dependent rapid *in situ* proliferation of local macrophages in Th2-biased inflammation accounts for extension of tissue alternatively activated macrophages (AAM), has challenged our prevailing understanding of a predominant monocytic contribution to augmented hepatic macrophage pool and sparked intensive debate (Jenkins et al., 2011; Tacke and Kurts, 2011). Indeed, under certain circumstances the intrahepatic macrophage infiltrate might actually be preferentially polarized towards the M2 phenotype (synonymous for AAM) as it has been observed in acetaminophen (APAP) treated mice by Holt et al. (2008). Congruently, these cells elicited a protective role by promoting inflammation resolution and tissue repair. Yet, as far as other experimental models of ALI are concerned, it is tempting to speculate that macrophage actions in these conditions are rather dominated by classically activated M1 macrophages (CAM) emanating from infiltrating monocytes. This hypothesis is supported by observations in acute carbon tetrachloride (CCl_4_) mediated liver injury in mice. Upon injury, the fraction of Ly6c^hi^ CD11b^+^ F4/80^+^ monocytes, representing the peripheral inflammatory monocyte subset, is significantly enlarged, whereas Ly6c^lo^ CD11b^+^ F4/80^−^ or Ly6c^lo^ CD11b^−^ F4/80^++^ cells, corresponding to either unconventional or resident macrophages, remain stable (Karlmark et al., 2009). Thus, the major body of evidence indicates that a large proportion of intrahepatic macrophages directly derives from blood-borne monocytes in conditions of experimental ALI. However, a thorough inspection of the nature of infiltrating macrophages is warranted, and it is likely that the paramount macrophage phenotype strongly depends on the respective injury model.

Monocyte migration into the liver is facilitated by a profound secretion of CCL2 (MCP-1) and presumably other chemokines by parenchymal and non-parenchymal liver cells. Liver macrophages represent a vigorous source of CCL2 (Karlmark et al., 2008). CCL2 is secreted and released into the systemic circulation inducing monocyte egress from the bone-marrow. However, available research activity has failed to elucidate a direct role of CCL2 in the transendothelial migration of monocyte into the abluminal liver compartment (Karlmark et al., 2009).

### Physiological stimuli trigger immune modulatory responses in KCs

Under homeostatic conditions KC response to stimulation with physiologically low levels of LPS is considered to restrict inflammation (Knolle and Gerken, 2000). Although KCs also secrete proinflammatory cytokines like TNF-alpha in the steady state context, this response may be too faint to elicit overt inflammation and is seemingly blunted by the concomitant release of anti-inflammatory signals. In fact, TNF-alpha secreted by KC can even contribute to dampen T-cell response (Knolle and Gerken, 2000). Various mechanisms are believed to render KCs to anti-inflammatory cells during homeostasis. Strikingly, KC activation in response to low LPS concentrations has profound impact on LSEC biology, emphasizing the close reciprocity between these two cell types. After low concentrated LPS challenge, hepatic macrophages secrete immunosuppressive IL-10, which acts in an autocrine manner on KC, but also paracrine on LSEC. IL-10 entails down-regulation of MHC-II and co-stimulatory molecules such as CD80 and CD86 on LSEC, thereby abrogating CD4^+^ T-cell activation (Knolle et al., 1998). Similar effects have been observed for prostanoids [i.e., Prostaglandin 2a (PGE2)] under non-inflammatory conditions (Knolle et al., 1998). Besides, naïve KC themselves are poor allostimulatory T-cell activators due to low expression of MHC-II, B7-1 (CD80), B7-2 (CD86) and CD40, and limit DC-induced antigen specific T-cell activation. This may represent another pathway of T-cell tolerance induction by KCs (You et al., 2008). Moreover, *in vitro* studies elucidated that IL-10 and prostanoids decrease expression of certain leukocyte adhesion molecules (ICAM-1, VCAM-1) on LSEC diminishing leukocyte endothelial transmigration into the liver parenchyma in homeostasis (Knolle and Gerken, 2000). Interestingly, KCs are also capable of disposing activated neutrophils invading the liver, thereby confining inflammatory processes (Bilzer et al., 2006). Phagocytosis of apoptotic neutrophil remnants by macrophages also attenuates production of proinflammatory cytokines like IL-1β and IL-8 in a TGF-β1, PGE2, and platelet-activating-factor (PAF) dependent fashion (Fadok et al., 1998). Accordingly, in a more recent publication it was demonstrated that KC priming with apoptotic splenocytes enhances production of IL-10 and reduces the release of proinflammatory signals (TNF-α and nitric oxide) through the Smad3 pathway after endotoxin challenge. Membrane-bound TGF-beta on apoptotic cells was reported to be the driving force of this phenomenon (Zhang et al., 2011). This is conclusive evidence that engulfment of apoptotic cell remnants by KCs favors tolerogenic immune response, as it has been also confirmed for “steady state” macrophages in general (Lucas et al., 2006; Chung et al., 2007). Efferocytosis of neutrophils has been demonstrated to induce IL-10 and TGF-beta production by macrophages and these cytokines are also closely linked to tissue repair (Ribeiro-Gomes et al., 2004; Filardy et al., 2010) Mechanisms of immune tolerance induction by quiescent KCs are illustrated in Figure 1.

### KC activation and its proinflammatory consequences upon acute liver insults

The response of KC to acute hazardous events contributes to hepatocyte killing directly and indirectly. Prelude of KC activation is the release of intracellular constituents from necrotic cells due to whatever cause (e.g., chemicals, physical, viral, hypoxia). The liberation of “danger” signals from inflamed, necrotic, or hypoxic parenchyma cells and the secretion of complement C5a directly contribute to KC activation. These endogenous damage-associated-molecular-pattern-molecules (DAMPs) bind to PRRs. The nuclear transcription factor high mobility group box-1 (HMGB-1) is a ligand of toll-like receptor 4 (TLR4) Its binding engages several downstream transcriptional factors (nuclear factor kappa B, NF-κB, AP-1, IRF-3, STAT-1) and kinases, eventually cumulating in upregulated synthesis of proinflammatory mediators with TNF-alpha being the most intensively studied mediator (Abu-Amara et al., 2010). Congruently, pharmacological inhibition of HMGB-1 by the triterpene glycyrrhizin ameliorated liver injury after ischemia-reperfusion in rats (Ogiku et al., 2011). Exogenous DAMPs (i.e., LPS) accumulating in the anhepatic phase of liver transplantation through translocation of intestinal microbes have been evidenced to play a role in TLR4 signaling in ALI as well and boost the inflammatory cascade (Fiorini et al., 2004). It can be concluded, that KCs are more sensitized to endotoxins in the presence of an additional hepatic insult, which lowers the threshold to recruit proinflammatory signals. Although considered a rather protective mechanism in homeostasis, engulfment of apoptotic hepatocytes can result in Fas ligand and TNF-alpha release by KC promoting tissue injury (Canbay et al., 2003), yet contradictory results in fulminant hepatitis exist (Zhang et al., 2011). After TNF-alpha binds to TNF-R1/TNF-R2 surface receptor on hepatocytes, downstream cascades via various domains (TRADD, FADD) imply activation of initiator caspases 8/9 and executioner caspases 3/6/7, which orchestrate cell death (Tacke et al., 2009). TNF-alpha and Fas (CD95) binding Fas ligand (FasL) expression are activated in fulminant hepatic failure by CD8^+^-cells, NK-cells, and KCs (Miyagawa-Hayashino et al., 2007; Malhi and Gores, 2008) and share common molecular pathways of instigation of apoptosis (Tacke et al., 2009). Furthermore, TNF-alpha can mediate caspase-independent launch of cellular death via formation of ROS and prolonged activation of JNK-pathway leading to extensive necrosis (Malhi and Gores, 2008). Concomitantly, TNF-alpha elicits protective antiapoptotic actions via NF-κB activation, resulting in transcription of survival genes (e.g., Bcl-xl, cFLIP). It is largely unknown which factors disarrange the balance of TNF-signaling from the predominate “prosurvival” side to the “proapoptotic” side in ALI (Malhi and Gores, 2008). Counterbalancing cytokines released by KCs include IL-4, IL-6, IL-10, and have shown to play a compensatory role in ALI by abrogating deleterious TNF signals, among others (Abu-Amara et al., 2010).

Another detrimental aspect of KC activation is secretion of various CC- and CXC-motif chemokines by activated KCs, which promote attraction of polymorphonuclear cells (neutrophil granulocytes), CD4^+^ T-cells, and monocyte-derived macrophages from the circulation to the hepatic microvasculature (Adams et al., 2010). When adhered to their target cell, granulocytes release ROS that trigger hepatocyte death and intensify resulting liver damage. This emphasizes the conception of heterogeneous cell types acting synergistically in ALI. CD4^+^ T-cell and neutrophil extravasation is facilitated by the induction of sinusoidal ICAM-1 and VCAM-1, interacting with the integrins CD11b/CD18 and CD29/49, through the KC-cytokines TNF-alpha and IL-6 (Sakamoto et al., 2002; Hanschen et al., 2008). Figure 2 summarizes by which mechanisms hepatic macrophages contribute to ALI.

## Monocyte Subpopulations Possess Distinct Phenotypes and Functions

### Monocytes are macrophage/dendritic cell progenitors and immune effector cells

Monocytes link the dramatic processes within the systemic and the hepatic compartment during ALI and failure by dictating systemic responses to local incidents and by providing hepatic macrophages that drive tissue injury. As to general features, monocytes are innate immune cells endowed with a broad panel of chemokine and PRRs ensuring their role as a potent antimicrobial and migratory leukocyte subset. They are able to identify a broad range of antigens and stimuli including dead cells, lipids, and bacterial pathogens (Geissmann et al., 2010). Based on their heterogeneity and versatility, monocytes exert multiple, occasionally opposed functions, and it is equivocal to which extent distinct monocyte subsets reflect diverging maturity stages (Gordon and Taylor, 2005; Geissmann et al., 2010). Lately, comprehensive phenotyping efforts have indicated that probably a continuum of phenotypes exists while the circumscribed profiles attributed to the defined subtypes merely constitute extreme polarizations (Wong et al., 2011). The capacity of rapidly releasing abundant amounts of proinflammatory cytokines (e.g., TNF-alpha, IL-1beta, IL-6, IL-8), reactive oxygen species, complement factors, and proteolytic enzymes upon various stimulatory events may render them as crucial contributors to the early systemic inflammatory response of acute and devastating systemic diseases. Opposite to this, the synthesis of immunosuppressive mediators (e.g., IL-10), which has been highlighted by numerous studies, can drive immune paralysis observed in ALI (Antoniades et al., 2008). Besides secretion of soluble factors, they augment the local macrophage pools following transendothelial migration. Moreover, they give rise to certain DCs (e.g., inflammatory TNF-alpha/iNOS-producing TipDCs) during inflammation (Tacke and Randolph, 2006) and repopulate tissue macrophages under physiological conditions (Klein et al., 2007).

### Development of blood monocytes from hematopoietic precursor cells

**Figure 3:**
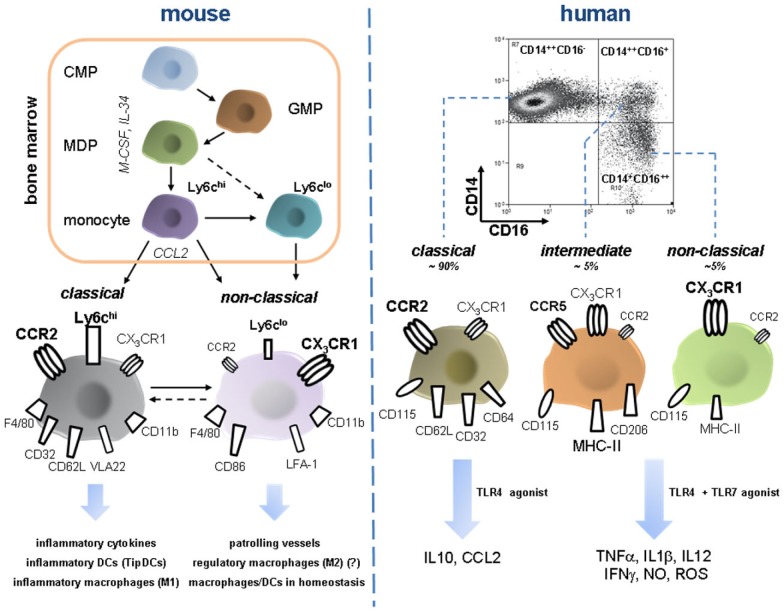
**Development and features of murine and human monocyte subsets**. Monocyte development from a common myeloid progenitor (CMP) occurs in the bone-marrow under the control of M-CSF and IL-34 and increasing lineage commitment. A macrophage dendritic progenitor (MDP) gives rise to Ly6c^hi^ monocytes and probably also to Ly6c^lo^ monocytes. Bone-marrow egress is directed by CCR2, maybe also by other pathways such as CXCR4/CXCL12 (not depicted). Peripheral Ly6c^hi^ can shuttle back to the bone-marrow and possibly transdifferentiate into Ly6c^lo^ monocytes, a process that might also occur in the periphery (e.g., in the spleen). Murine monocyte subsets constantly express F4/80 and CD11b. Classical Ly6c^hi^ are characterized by high expression of CCR2 but only moderate levels of the fractalkine receptor CX_3_CR1. Furthermore, they express several scavenger receptors (e.g., CD32), selectin ligand CD62L, and the integrin VLA-2. Ly6c^hi^ monocytes have a proinflammatory profile since they secrete inflammatory cytokines, migrate into inflamed tissue and give rise to inflammatory macrophages (sharing qualities with classical M1 macrophages), and DC subsets. Ly6c^lo^ are less abundant in the periphery and express high CX_3_CR1, CD86, and LFA-1 whereas CCR2 is almost absent. They patrol blood vessels by crawling across endothelium. During inflammation they can rapidly or protracted enter tissue and presumably develop into macrophages with a rather anti-inflammatory phenotype eliciting wound repair (reminiscent of M2 macrophages). In homeostasis they are currently designated as precursor cells of local macrophages and dendritic cells. Importantly, Ly6C^hi^ derived macrophages can very likely also acquire anti-inflammatory phenotypes in peripheral tissues such as the liver. Intermediate Ly6c^int^ are not depicted here. FACS dot plot in the right upper corner illustrates characteristic distribution of human monocyte subsets. CCR2 high expressing CD14^++^CD16^−^ monocytes are termed classical monocytes, representing the vast majority of circulating monocytes. Despite phenotypic homology to murine inflammatory Ly6c^hi^ monocytes they release immune suppressive IL-10 upon LPS stimulation. In turn, intermediate and non-classical monocytes that are defined by the expression of CD16, display moderate to high CX_3_CR1 and varying density of CD14 and are far less abundant, but release various inflammatory cytokines after challenge with TLR4 and TLR7 agonists. These functions sharply contrast the rather anti-inflammatory phenotype of Ly6c^lo^ monocytes as their putative murine counterparts. Intermediate monocytes and classical monocytes have a distinct surface protein profile (e.g., CCR5 is almost exclusively expressed on the intermediate subset) though functional differences are not fully defined. Abbreviations: CMP, common myeloid progenitor; GMP, granulocyte myeloid progenitor; IFNg, interferon gamma; LFA-1, lymphocyte function antigen 1; M-CSF, macrophage colony-stimulating factor; MDP, macrophage dendritic cell progenitor; NO, nitric oxide; TipDCs, TNF–iNOS-producing dendritic cells; VLA-2, very late antigen 2.

Monocytes circulate in blood vessels, spleen, and bone-marrow (Geissmann et al., 2010). Their development entails several precursor cells ranging from a pluripotent hematopoietic stem cell over a common myeloid (CMP) to a macrophage/DC progenitor (MDP) along a path of rising lineage restriction and commitment encompassing additional intermediate stages (Robbins and Swirski, 2010). This process is under control of the growth factors Csf-1 (alternatively termed M-CSF; Kawasaki et al., 1985) and IL-34 (Lin et al., 2008) that both bind to Csf-1R (M-CSFR/CD115; Wei et al., 2010) and synergistically govern monocyte differentiation in a non-redundant manner (Geissmann et al., 2008). CMPs are referred to as Lin^−^Sca^−^IL-7Ra^−^CD117^low^CD34^+^CD16^+^ cells that exhibit CD115 and CX_3_CR1 on their surface and differentiate into macrophages, DCs, and monocytes but not into granulocytes (Akashi et al., 2000; Fogg et al., 2006; Varol et al., 2007). Development from MDP into monocytes occurs in the bone-marrow as the last step of monocyte differentiation before they exit into circulation. This event that is mainly directed by CCR2/CCL2 interactions (especially upon systemic inflammation), but may involve other homeostatic pathways such as CXCR4/CXCL12 (Serbina and Pamer, 2006; Geissmann et al., 2008; Figure 3). The prevalent paradigm of the bone-marrow as the sole source of monocytes, however, has been challenged by more recent investigations. According to these findings, a certain fraction of circulating monocytes delineates from a splenic reservoir in the cords in the subcapsular red pulp and does not require CCR2 signals to enter systemic circulation (Swirski et al., 2009). In fact, release from the splenic site relies on factors including angiotensin II (Swirski et al., 2009). Concordantly, angiotensin-II-lowering agents like angiotensin-converting-enzyme inhibitors confined the mobilization of splenic monocytes in a model of myocardial infarction (Leuschner et al., 2010). CCR2-independent non-medullar monocyte resources therefore have to be incorporated in further studies dedicated to intervene at monocyte allocation in pathological settings.

### Phenotype and function of murine “classical” monocytes

Irrespective of their human or murine origin, peripheral monocytes are defined by expressing the pan-monocytic markers CD115/CX_3_CR1 and they lack expression of surface molecules such as Nkp-46, CD3, CD19, or CD15 that characterize NK-cells, T-cells, B-cells, and neutrophils, respectively (Auffray et al., 2009). In mice discrimination of CD11b^+^F4/80^+^ monocyte subtypes relies on the differential expression levels of Ly6C which is recognized by the antibody RB6-8C5 (Gr1; Geissmann et al., 2003; Taylor and Gordon, 2003). Owing to a deficit of functional data, further separation of murine monocyte subsets on the grounds of variable CD43 expression, as it has been proposed in the current nomenclature (Ziegler-Heitbrock et al., 2010), will be neglected here. Ly6c^hi^ (Gr1^hi^) monocytes constitute comparatively big cells that are distinguished by CCR2^hi^CX_3_CR1^lo^ expression and the presence of VLA-2 and CD62L adhesion molecules (Taylor and Gordon, 2003; Robbins and Swirski, 2010; Figure 3). Representing the major subpopulation, they are designated “inflammatory” or “classical” monocytes due to the extensive capacity of secreting proinflammatory mediators (i.e., TNF-alpha, iNOs, IL-12, type 1 interferon) and migrating into inflamed tissues, as it has been demonstrated for inflamed peritoneum, skin, and infarcted myocardium (Gordon and Taylor, 2005; Tacke and Randolph, 2006; Robbins and Swirski, 2010). If activating inflammatory signals hold off, egressed Ly6c^hi^ monocyte shuttle back to the bone-marrow and down-regulate CCR2 expression. Upon inflammatory stimuli including ALI, Ly6c^hi^ monocytes massively translocate from bone marrow into the circulation and decrease again as the inflammation abates (Karlmark et al., 2009; Robbins and Swirski, 2010). Subsequent to tissue entry, “classical” monocytes give rise to macrophages and inflammatory DCs including TipDCs after MyD88-pathway activation and hence endorse microbial killing (Strauss-Ayali et al., 2007; Serbina et al., 2008). Likewise, accumulation of these cells also occurs in non-infectious inflammation settings (Nahrendorf et al., 2007; Robays et al., 2007; Tacke et al., 2007). In the chronically inflamed liver, Ly6c^hi^ monocytes acquire a phenotype reminiscent of CAM thereby perpetuating the intrahepatic inflammatory milieu that promotes fibrogenesis (Karlmark et al., 2009; Tacke and Kurts, 2011). The concept of the progeny of M1 macrophages from Ly6c^hi^ monocytes bases on observations on mucosal TNF-alpha- and iNOs-producing macrophages in toxoplasma gondii infection (Dunay et al., 2008).

### Characteristics of “non-classical” monocytes in mice

In contrast to “classical” monocytes, the functional spectrum of the less frequent murine Ly6c^lo^ (Gr1^−^) “non-classical” or “resident” monocytes has not been sufficiently clarified. Controversial reports have hampered the formation of a clear concept for this cell type. Some experimental evidence indicates that Ly6c^lo^ monocytes originate from Ly6c^hi^ cells during maturation (Sunderkotter et al., 2004; Tacke et al., 2006; Varol et al., 2007), some of the Ly6c^lo^ monocytes, however, develop independently in the bone-marrow (Geissmann et al., 2003; Hanna et al., 2011). It is well acknowledged, that “non-classical” monocytes have the potential to home to uninflamed tissue in a G-protein-dependent manner and thus renew local macrophages and DCs, whereas tissue inflammation does not induce Ly6c^lo^ monocyte recruitment in early phases of inflammation (Geissmann et al., 2003). Furthermore, their human counterpart cells (CD16^+^ monocytes) were capable of reversely transmigrating across unstimulated human umbilical vein endothelial cells (HUVEC), a process that involved the adoption of a DC-like profile (Randolph et al., 2002). Consecutive investigations, however, indicated that Ly6c^lo^ monocytes constantly crawl in the lumen of the blood vessels across the endothelial cell layer, mediated by the integrin LFA-1 and CX_3_CL1, and sense the subjacent space for danger signals (Auffray et al., 2007). Yet, when manifest inflammatory processes are absent, Ly6c^lo^ monocytes exhibited very low tendency to extravasate. In turn, during acute *Listeria monocytogenes* infection patrolling “non-classical” monocytes rapidly transmigrate toward the local irritant stimulus and transiently produce inflammatory cytokines (Auffray et al., 2009). As inflammation evolves, Ly6c^lo^ then might differentiate into alternatively activated (M2) macrophages that suppress inflammation and convey repair, angiogenesis, and wound healing (Nahrendorf et al., 2007; Auffray et al., 2009). CCR2 expression of murine Ly6c^lo^ is low to absent in agreement with a rather anti-inflammatory profile. Instead, they express abundant CX_3_CR1, which might potentially explain their longer live-span, and express different adhesion molecules (high LFA-1 expression) in comparison to the “classical” counterpart (Figure 3).

### Divergent properties of human monocyte subtypes

Despite increasing knowledge about phenotypic and genetic congruency between murine and human monocyte subsets (Ingersoll et al., 2010), there seem to be profound functional disparities between monocytes in different species. In addition, some differences on the phenotype level are present as well that hinder the experimental analysis of monocytes in acute murine liver injury. For instance, differential regulation of monocytic HLA-DR expression in ALI cannot be studied in mice since HLA-DR is not present on circulating monocytes in that species. By now the distinction of three different human monocyte subpopulations has been implemented in the official nomenclature (Ziegler-Heitbrock et al., 2010; Wong et al., 2011; Zawada et al., 2011). Accounting for approximately 90% of circulating monocytes, human “classical” monocytes are CD14^++^CD16^−^ cells with high levels of CCR2 and low levels of CX_3_CR1 thereby closely resembling murine Ly6c^hi^. They feature relatively high CCR1, CXCR1, CXCR2, CXCR4, CD32, CD62L and CD64 expression but only low levels HLA-DR and have significant phagocytic activity (Wong et al., 2011; Zimmermann et al., 2011). Following endothelial transmigration, these cells tend to persist in the subendothelial space and develop into macrophages whereas CD16 positive monocytes are predisposed to develop into migratory DCs with T-cell-stimulatory capacity (Randolph et al., 2002). Monocytes that display CD16 on their surface are now further divided into an “intermediate” subset (with maintained high expression of CD14, therefore termed CD14^++^CD16^+^) and “non-classical” CD14^lo^ cells with equal to increased amounts of CD16 (CD14^+^CD16^++^; Figure 3). Some striking discrepancies exist between the latter subsets. “Intermediate” monocytes exhibit highest HLA-DR, mannose receptor CD206 [a M2 marker (Zimmermann and Adams, unpublished data)] and CCR5 levels, whereas the fractalkine receptor is most abundant on the CD14^lo^ subtype (Wong et al., 2011; Zawada et al., 2011). Selective CD206 expression by CD14^++^CD16^+^ cells might refer to their preferential capacity to give rise to AAM, though this needs functional confirmation. Both subsets show comparable levels of CD32 and only marginal CCR2 expression (Zawada et al., 2011). Functional differences between the CD16^+^ monocytes are still somewhat ill-defined and the majority of available functional data does not distinguish between the various subtypes. “Intermediate” monocytes seem to be specifically involved in HIV infection, possibly because of their CCR5 expression that confers viral cell-entry in CCR5-tropic-HIV-1-(M)-strains (Jaworowski et al., 2007). The prominent feature of CD16^+^ monocytes to release proinflammatory mediators upon *in vitro* stimulation with LPS has been attributed to the CD14^hi^ expression subset (Grage-Griebenow et al., 2001), whereas, according to another study, CD14^lo^ monocytes show only weak phagocytic activity and secrete only low amounts of cytokines and reactive oxygen species after LPS exposure but synthesize TNF-alpha, IL-1beta as well as CCL3 subsequent to viral and nucleic acid stimuli involving activated TLR7–TLR8–MyD88–MEK pathway (Cros et al., 2010). Similar to murine Ly6c^lo^ monocytes, these cells patrol in the blood vessels in an integrin-dependent manner (Cros et al., 2010).

### Expansion and function of CD16 expressing monocytes during human diseases

Regardless of the heterogeneity of CD16^+^ monocytes, there are a multitude of pathological conditions leading to an expansion of the minor CD16^+^ subset. Increased proportions of CD16^+^ monocytes in “sterile” inflammation have been reported in rheumatoid arthritis (Kawanaka et al., 2002; Wijngaarden et al., 2003), hemodialysis (Nockher and Scherberich, 1998), and atherosclerosis/coronary artery disease (Rothe et al., 1996; Schlitt et al., 2004), among others, and frequently mirrored disease activity and severity. Infectious diseases such as HIV (Thieblemont et al., 1995), erysipelas (Horelt et al., 2002), and bacterial sepsis (Fingerle et al., 1993) also exhibit a substantial rise in CD16^+^ monocytes. Interestingly, peripheral counts of this subset were pronounced in patients with Gram-negative bacteremia (Herra et al., 1996), which is highly prevalent in patients with ALF. Resolution of inflammation and successful antimicrobial treatment result in decline of peripheral monocytes (Horelt et al., 2002). In the context of liver disease, a shift in distribution of peripheral monocytes and monocyte-derived hepatic macrophage subsets toward the CD16^+^ subtype indicates disease progression and is highest in patients with end-stage Child C cirrhosis (Zimmermann et al., 2010). Furthermore, under constant flow mimicking sinusoidal shear stress CD16^+^ monocytes are more prone to transmigrate across TNF-alpha/IFN-gamma stimulated hepatic sinusoidal endothelium than their CD16^−^ counterpart (Liaskou et al., unpublished data). It is obvious to anticipate that CD16^+^ monocytes also expand in ALF, yet, investigations in this subject are still pending. The total number of monocytes harshly decline in acetaminophen-induced ALF (AALF), but the relative distribution of monocyte subsets was not addressed in this study (Antoniades et al., 2012).

In sharp contrast to their putative murine counterparts, CD16^+^ monocytes are tagged “proinflammatory” since *in vitro* they are potent producers of proinflammatory mediators such as TNF-alpha, IL-12, IFN-gamma, CCL3, CCL4, CXCL9, and nitric oxide after stimulation with either TLR2-/TLR4-agonists, tumors or even spontaneously. They hence might pivotally contribute to systemic enhancement of the proinflammatory environment in ALF (Belge et al., 2002; Szaflarska et al., 2004; Zimmermann et al., 2010). Vice versa, human CD16^−^ monocytes secrete immunosuppressant IL-10, which is nearly absent on protein as well as transcriptional level in the CD16^+^ subset. They thus clearly feature anti-inflammatory traits (Mizuno et al., 2005; Zimmermann et al., 2010; Figure 3). Of note, the profound discrepancies between similar phenotypic features and functional disparities between murine and their putative homolog human monocyte subpopulations is beyond clarification. In addition, the biological relevance of proinflammatory qualities attributed to human CD16^+^ monocytes has not been completely uncovered but future scientific studies will certainly help to elucidate open questions in that controversy.

## Monocytes/Macrophages in Experimental Models of Acute Liver Injury

Given the heterogeneous nature of the various triggers of ALI as a common sequela, it is a rational approach to use different experimental models in order to shed light on the pathogenesis of this disorder. Despite a broad panel of different models of ALI in rodents and other mammals, there are currently no experimental models that appropriately reproduce all aspects of acute human liver injury/failure (Newsome et al., 2000). Importantly, the diagnostic King’s college criteria for ALI and ALF in human mainly incorporate biochemical and clinical deteriorations that are scarcely applicable to (small) animals (Tunon et al., 2009). Despite prevailing limitations, animal models have unraveled relevant aspects of macrophage action in this devastating disease condition. Selected models will be summarized in this paragraph. Approaches that aimed at ablating monocyte/macrophage migration and function by targeting chemokine pathways are discussed in subsequent sections.

### Experimental models of ALI involving chemical substances

Chemical models of ALI comprise substances such as acetaminophen (APAP), galactosamine and LPS (GalN–LPS), the plant-derived lectin Concanavalin A (ConA), double-stranded RNA (polyI:C), alpha-Galactosylceramide (alpha-GalCer), single endotoxins, carbon tetrachloride (CCl_4_), CpG, DMSO, CD40L, amanitin, and thioacetamide, but only very few have been used to substantially unravel role and mechanisms of macrophage activity in this disease complex (Wu et al., 2010).

#### Concanavalin A

Concanavalin A (ConA) is a plant mitogen with carbohydrate-binding (lectin) properties extracted from *Canavalia ensiformis*, which causes a vigorous CD4^+^ T-cell stimulation resulting in hepatic TNF-related hepatic necrosis after single administration (Mizuhara et al., 1994). Initial evidence for a causative role of KCs in ALI following ConA challenge stems from a study involving gadolinium-chloride-(GdCl_3_)-treatment (Okamoto et al., 1998). GdCl_3_ reduces KCs and compromises their function, though a complete depletion is not achieved (Michael et al., 1999). Reduction/alteration of KCs yielded less damage, though intrahepatic TNF-alpha expression was not changed (Okamoto et al., 1998). Based on a similar strategy of KC manipulation, liver injury could be markedly ameliorated, accompanied by reduced intrahepatic cytokine levels (including TNF-alpha) and diminished infiltration of CD4^+^ T-cells, in another study (Morita et al., 2003). Schumann et al. (2000) could demonstrate that KC elimination by clodronate-loaded liposomes significantly restricted the spreading of focal confluent necroses. This phenomenon was attributed to KC-dominant TNF-alpha secretion in that model. However, systemic levels of TNF-alpha were not affected and injection of soluble rmuTNF could not abolish the protective effect of KC-ablation (Schumann et al., 2000). The latter observation suggests that membrane-bound TNF-alpha is pivotal in driving ConA-induced liver injury. The authors could also observe a harsh decline of systemic IL-6 levels, a cytokine which has hepatoprotective qualities and governs liver regeneration (Streetz et al., 2003; Tacke et al., 2009). This might appear contradictory to the blunted liver damage observed here, since IL-6-deficient mice were more susceptible to ConA-mediated fulminant hepatitis than wild-type animals (Tagawa et al., 2000). Succeeding investigations underscored the relevance of KCs for the contribution to a dominant Th1 immune response in the ConA model (Chen et al., 2011). Of note, several studies demonstrated that repetitive sublethal ConA injections induced immunotolerance, which could be attributed to instigation of IL-10 production by KCs acting synergistically with regulatory T-cells (Erhardt et al., 2007; Erhardt and Tiegs, 2010). Mechanistically, inhibition of NF-κB activation in KCs by intraportally injected decoy oligonucleotide-loaded gelated particles during ConA hepatitis but not ischemia/reperfusion related liver injury led to diminished TNF-alpha production and reduced phosphorylation of the proapoptotic jun N-terminal kinase (JNK), that essentially drives hepatocyte apoptosis (Hoffmann et al., 2009).

#### d-galactosamine/lipopolysaccharide

The coadministration of d-galactosamine and LPS (GalN/LPS) is a model of synchronous liver injury and endotoxin-shock and is governed by monocyte/macrophage-released TNF-alpha that essentially induces hepatocyte apoptosis (Josephs et al., 2000; Stuart et al., 2011). In accordance to the ConA model described precedingly, selective pharmacological inhibition of the NF-κB downstream cascade in KCs abrogated TNF-alpha synthesis and blunted liver damage (Hoffmann et al., 2009). TNF-alpha release by KCs in this model is negatively modulated by the Ron receptor tyrosine kinase and Lys-Cre Ron TK^ft/ft^ mice with a conditional myeloid-specific Ron deletion featured exacerbated damage and increased lethality in response GalN/LPS induced injury (Stuart et al., 2011). Ron deficient mice correspondingly had higher *Tnf-alpha* gene transcription (Stuart et al., 2011).

#### Acetaminophen (APAP)

In terms of clinical relevance and dose-dependent toxicity, APAP meets the criteria for a suitable model of ALF (Newsome et al., 2000). Following exposure to toxic doses, physiological detoxifying pathways (glucuronidation, sulfation, renal excretion) are depleted and acetaminophen enters Cytochrom-P450 metabolism, which in turn yields abundant production of the toxic metabolite *N*-acetyl-*p*-benzoquinoneimine (NAQI; Corcoran et al., 1980). Centrilobular necrosis and fulminant organ failure emerge when protective glutathione levels are exhausted (Roberts et al., 1990). This is significantly accelerated during concomitant exposure to cytochrom-P450-inducing drugs and alcohol. Covalent binding and arylation of important cell proteins by NAQI results in loss of protein function and profoundly impairs organelle and cell integrity (Cohen and Khairallah, 1997). Hepatic macrophages contribute to hepatotoxicity via different mechanisms encompassing the formation of nitric oxide and superoxide that react together to produce peroxynitrite, which again exhibits hydroxyl radical-like activity (Michael et al., 1999). Importantly, KCs release a multitude of inflammatory cytokines, in particular TNF-alpha, after toxic APAP exposure (Laskin et al., 1986). In line, earlier studies reported that GdCl_3_ treatment prior to APAP challenge in mice significantly attenuated liver damage and associated mortality (Blazka et al., 1995; Laskin et al., 1995; Michael et al., 1999). Subsequent series of experiments employing clodronate-loaded liposomes, a strategy that enables more profound KC depletion than GdCl_3_, showed contradictory results. Pretreatment with clodronate liposomes led to almost complete KC depletion, decrease of intrahepatic immune modulatory cytokines such as IL-6, IL-10, or IL-18, and markedly enhanced susceptibility to acetaminophen (Ju et al., 2002). IL-10 was demonstrated to diminish APAP-associated hepatic necrosis and lethality (Bourdi et al., 2002). In support of this work, Holt et al. (2010) found that KC-dependent maintenance of liver sinusoidal endothelial cell integrity is another contributory factor to hepatoprotection during acetaminophen toxicity. Ambiguously, a protective role of KCs was not reproducible in a successive study also using clodronate liposomes (Campion et al., 2008). Conclusively, the precise nature of the functional dichotomy of hepatic macrophages in APAP toxicity is yet to be elucidated. A more recent study portended that opposing roles of hepatic macrophages might be attributable to cell origin since prolonged infiltration of monocyte-derived macrophages clearly promoted resistance to APAP damage (Holt et al., 2008). The concept of sequential immigration of different macrophage subpopulations was also evidenced in acute thioacetamide-mediated injury (Mori et al., 2009), in which KC-inactivation alleviates the extent of liver deterioration and accelerates liver regeneration (Andres et al., 2003; Bautista et al., 2010). Of note, beneficial properties of recently infiltrated macrophages were note reproducible in the carbon tetrachloride (CCl_4_) model of acute injury (Karlmark et al., 2009; Mitchell et al., 2009).

#### Carbon tetrachloride (CCl_4_)

CCl_4_ is widely employed in liver research as it reliably induces an acute toxic liver injury and even liver fibrosis within several weeks after repetitive injection. Cessation of CCl_4_ administration after single and long-term use allows the study of injury regression (Berres et al., 2010; Karlmark et al., 2010). Acute hepatic toxicity is a consequence of carbon-tetrachloride-induced formation of a highly reactive carbon-entered trichloromethyl radical through cytochrom-P450 isoenzymes that interacts with hepatic proteins as well as lipids and deteriorates cellular membranes (Luckey and Petersen, 2001). Rapid centrilobular damage succeeds already after one injection. CCl_4_-exposure entails upregulation of *Tnf-alpha*, *Il-1beta*, *Il-6*, *Tgf-beta*, and *cyclooxygenase-2* mRNA transcripts and synthesis of reactive oxygen species plus eicosanoids in KCs (Alric et al., 2000; Luckey and Petersen, 2001). However, mechanistic evidence for a relevant role of KCs in CCl_4_-mediated liver injury is scarce. Transient elimination of blood monocytes and hepatic macrophages cells prior to toxin-injection did not alter the magnitude of resulting liver damage, suggesting that monocytes/macrophages do not endorse acute liver damage in that specific model, but rather modify the subsequent inflammatory response within the injured liver. These findings were corroborated by data from chemokine-directed targeting of infiltrating macrophages in the same work (Karlmark et al., 2009). Basically, the secretory profile of hepatic macrophages might implicate both harmful and beneficial effects for the hepatic outcome of CCl_4_-mediated injury, since it has been shown that disruption of TNF-alpha-pathways partially restores liver integrity (Morio et al., 2001), whereas mice lacking the gene for nitric oxidase synthase (NOS II) or IL-6 are more susceptible to liver damage in that model (Morio et al., 2001; Bansal et al., 2005). Noteworthy, the reliability of CCl_4_ for studying pathogenesis of ALI is hampered by the considerable inherent variability of this model related to differences in species, age, and development of the metabolizing cytochrome-P-450 system (Newsome et al., 2000).

### KC in the molecular cascade of ischemia/reperfusion as a surgical model of ALI

Acute liver damage is an important sequel to hepatic ischemia/reperfusion (I/R) injury as it occurs in the clinical settings of trauma, shock of any cause, transient surgical interruption of hepatic blood flow and liver transplantation (Howard et al., 1990). A vast array of I/R animal studies have highlighted the role of inflammatory cytokines, reactive oxygen species, and sequestration/activation of leukocytes in the self-amplified cytotoxic cascade entailing the demise of liver parenchyma (Zhai et al., 2011). During I/R injury, KC swelling as a consequence of ion channel breakdown due to the depletion of adenosine triphosphate (ATP) contributes to devastating hepatic microcirculatory dysfunction. Moreover, complement factors and lymphocyte-released IFN-gamma are strong activating stimuli for KCs (Wanner et al., 1996). NADPH oxidase activation in KCs has been shown to be an integral component in the postischemic injury phase (Jaeschke, 2003). They secrete critical amounts of TNF-alpha, IL-1beta, promigratory chemokines, and free radicals. These humoral factors and reactive species directly promote hepatocyte death, sinusoidal endothelial damage and favor leukocyte adhesion, and infiltration, all of which processes propagate I/R injury (Abu-Amara et al., 2010). Monocyte-derived macrophages enter the liver in the rather late postischemic phase and sustain the inflammatory milieu responsible for continuous destruction of local tissue (Zhai et al., 2011). In the scope of ongoing liver inflammation due to I/R injury, KCs are able to change from a proinflammatory to a more immune modulatory phenotype by releasing IL-10 (Bamboat et al., 2010; Ellett et al., 2010).

In agreement with the well-described inflammatory properties of hepatic macrophages in that model, many investigators observed that KC blockade improves ischemic liver injury (Hardonk et al., 1992; Giakoustidis et al., 2003, 2006; Tomiyama et al., 2008). Ellett et al. (2010) demonstrated an imbalanced cytokine milieu in the absence of KC in I/R combined with bowel congestion, presumably owing to a lack of protective IL-10. This was associated with largely enhanced liver injury and increased mortality. Beneficial effects of hepatic macrophages during ischemic insults could be attributed to liver resident but not infiltrating macrophages in a previous paper, that elegantly delineated the differential contribution of resident and infiltrating macrophages by using clodronate liposomes in CD11b diphtheria toxin receptor (DTR) mice. The KC-specific antioxidant heme-oxygenase was described to be protective in this model (Richards et al., 2010). Conflicting results regarding KC function in I/R injury may be explained by substantial methodic differences in KC depletion in the pertinent studies (e.g., GdCl_3_ vs. clodronate liposomes) or by the fact that the different observations merely depict one extreme of the diametrically opposed biphasic functions of hepatic macrophages in ALI. In conclusion, the net effect of KC activity in I/R injury remains to be clarified.

## Monocyte and Macrophage Related Chemokine Pathways in Acute Liver Injury

### Classification of the chemokine system

Chemokines belong to a family of heparin-binding small promigratory cytokines that orchestrate the trafficking of immune cells, which is indispensable for immune cell functions in homeostasis (recirculation of leukocytes to secondary lymphoid tissues) and inflammation. They have received tremendous attention throughout the last years in terms of their function in liver inflammation and resulting fibrosis and many studies have investigated whether modifications of chemokine pathways could imply therapeutic benefits (comprehensively reviewed in Zimmermann and Tacke, 2011). Apart from CX_3_CR1 and CCR2 monocyte differentially express CCR1, CCR5, CCR8, CXCR1, CXCR2, and CXCR4 that may all influence their migratory fate to varying degrees (Gordon and Taylor, 2005; Zawada et al., 2011; Zimmermann and Tacke, 2011).

### CCL2 (monocyte-chemoattractant-protein-1) in acute liver injury

CCL2 (monocyte-chemoattractant-protein-1, MCP-1) is the pivotal ligand of CCR2 and has been intensively studied in the context of liver injury. Many intrahepatic cell subsets release CCL2 upon a deleterious stimulus encompassing hepatocytes (Dambach et al., 2002) and non-parenchymal cells such as KCs (Jaeschke and Smith, 1997; Marra et al., 1998; Dambach et al., 2002; Seki et al., 2007), biliary epithelial cells (Marra et al., 1998; Tsuneyama et al., 2001; Kruglov et al., 2006; Harada et al., 2011), and quiescent or activated HSC (Marra et al., 1993, 1995, 1998) supporting the notion that CCL2-release represents an ubiquitous inflammatory pathway that is highly conserved in cells from entirely distinct ontogenic backgrounds. In the context of ALI, CCL2 as well as other proinflammatory chemokines such as CCL3 (MIP-1alpha), CCL4 (MIP-1beta), CXCL9 (MIG), and CXCL10 (IP-10) are secreted in response to TNF-alpha, IL-1alpha, IL-1beta, LPS, and reactive oxygen species stimuli originating from damaged hepatocytes, KCs, intestinal bacteria, and other sources (Czaja et al., 1994; Heymann et al., 2009). Interestingly, hepatic upregulation of CCL2 marks a very early event in the course of acute hepatic damage, since it is detectable in the first 1–4 h after the onset of CCl_4_-induced liver injury (Czaja et al., 1994; Marra et al., 1999; Leifeld et al., 2003; Karlmark et al., 2009). First evidence of the relevance of CCL2 in ALI delineate from a study involving CCl_4_- and galactosamine exposed rats, that displayed marked increase of CCL2 (Czaja et al., 1994). Apart from CCl_4_-mediated ALI, induction of CCL2 expression has been observed in GalN/LPS (Leifeld et al., 2003), thioacetamide (Mori et al., 2009), ConA (Ajuebor et al., 2003; Leifeld et al., 2003), and drug-(APAP)-induced liver toxicity (Dambach et al., 2002). In human ALF increased CCL2 levels have been detected in various studies (Leifeld et al., 2003; James et al., 2005; Roth et al., 2009; Antoniades et al., 2012). Of note, CCL2 serum concentrations in a pediatric study cohort correlated with clinical disease severity, transaminase levels, and coagulopathy (James et al., 2005) and highest CCL2 levels were associated with fatal outcome in patients with acetaminophen-induced liver failure (Antoniades et al., 2012). Functional cues for the importance of increased intrahepatic chemokines for the hepatopetal trafficking of human monocytes/macrophages in ALF arise from one study that used homogenized liver samples from ALF patients livers containing elevated amounts of CCL2. Therein, a chemotactic stimulus on monocytes could be observed (Leifeld et al., 2003). Chemotaxis was significantly decreased by simultaneous treatment with CCL2, CCL3, CCL4, and CCL5 neutralizing antibodies. The latter chemokines were comparably elevated in acute human liver failure specimen suggesting that they also might contribute to the monocyte/macrophage attraction in this disease (Leifeld et al., 2003).

### CCR2 in acute liver injury

Experimental studies involving rodents with genetic CCR2 deficiency have shed light onto the functional relevance of the CCR2/CCL2 axis for the influx of monocytes/macrophages in ALI. In a study performed by our group, wild-type mice exhibited a vigorous influx of CD11b^+^ F4/80^+^ macrophages detectable 4 h and peaking at 48 h post-CCl_4_-administration. Macrophage infiltration paralleled tissue and hepatic CCL2 upregulation suggesting that CCL2/CCR2 instructed macrophage-tissue-ingress. This was corroborated by the mitigation of CD45^+^ cell and macrophage accumulation in the liver in *Ccr2*^−/−^ knockout mice occurring 4 h after toxin-challenge (Karlmark et al., 2009). Since CCL2 mainly controls monocyte bone-marrow egress, levels of circulating monocytes declined as well, yet to a lesser extent. Mitchell et al. (2009) also detected diminished proportions of CD11b^+^ F4/80^+^ macrophages as well as their respective blood precursors but not CD11c^+^ cells post-CCl_4_-challenge in *Ccr2* deficient mice at an early time-point. In contrast to the previous work, hepatic damage was reduced in the latter study, as expressed by lower aminotransferases. This suggests a detrimental role of CCR2^+^ infiltrating macrophages in the setting of acute liver damage. Furthermore, the critical upregulation of the proinflammatory cytokines and chemokines TNF-alpha, IL-1beta, IL-6, CCL2, CCL3, CXCL9, and CXCL10 was markedly attenuated, which might provide a possible mechanism for the observed decrease in liver injury. In addition, this observation implies that invading macrophages are an important source of inflammatory mediators and thereby directly exacerbate tissue harm. Despite this, the putative disadvantageous effects of CCR2-controled macrophage influx observed here cannot be readily extrapolated from the carbon tetrachloride model to other injury settings. Although data obtained from various studies involving acetaminophen-challenged CCR2^−/−^ mice confirmed that genetic disruption of CCR2 yields less infiltration of macrophages (Dambach et al., 2002; Holt et al., 2008), CCR2 deletion did not limit hepatic necrosis (Dambach et al., 2002) but effectuated increased liver damage with amplified apoptosis (Hogaboam et al., 2000). Noteworthy, Dambach et al. (2002) reported augmented serum CCL2 levels and induction of TNF-alpha in knockout animals in contrast to the CCl_4_ model. These findings support the assumption that immigrating CCR2^+^ macrophages in APAP-induced ALI convey their hepatoprotective effect via resolution of inflammation (e.g., through induction of neutrophil apoptosis) and direct necrosis regression due to phagocytosis of cell debris. In line, the induction of several hallmark genes of a M2 macrophage phenotype (*Ym1, Fizz1, Arg-1*) associated with tissue repair and wound healing in infiltrating macrophages has been reported (Holt et al., 2008). Concordantly, clearance of necrotic areas in *Ccr2*^−/−^ animals was substantially delayed in the reparation phase. The controversy regarding the consequence of disruption of the CCR2/CCL2 axis in acute liver toxicity has been further substantiated by a study in which CCL2-deficient mice were characterized by delayed necrosis formation, ameliorated liver enzyme profile, extenuated elevation of TNF-alpha, lymphotoxin-beta, and reduced oxidative stress after single gastric CCl_4_ administration (Zamara et al., 2007). These antithetic results suggest CCR2-independent effects of CCL2 which have been described in *in-vitro*-assays before (Schecter et al., 2004; Kruglov et al., 2006). The proposed protective role of CCL2 in acute injury settings recapitulates earlier findings indicating that CCL2 neutralization caused enhanced mortality in a murine model of lethal endotoxemia (Zisman et al., 1997). A monocyte-independent mechanism of CCL2-related hepatoprotection in acute T-cell mediated hepatitis has been provided by Ajuebor et al. (2003) demonstrating that CCL2 also directly hinders IL-4 production by NKT cells.

Importantly, biological effects of CCL2 deficiency in liver injury may be reverted by the compensatory upregulation of CCL8 (MCP-2) and CCL7 (MCP-3), which also bind to CCR2 and mediate monocyte recruitment to the liver in *L. monocytogenes* infection (Jia et al., 2008). Furthermore, monocyte/macrophage trafficking to the site of hepatic injury might also occur without the involvement of chemokines (Shi et al., 2010). Despite these possible confinements, intriguing data from murine steatohepatitis have given proof of the generic feasibility and efficacy of CCL2 antagonism by small molecules in combating liver diseases (Baeck et al., 2012), Clinical trials involving CCR2 antagonists in non-liver disease entities are already at hand. Pharmacological inhibition of CCL2 by *Spiegelmer* technology resulted in a pronounced decrease of monocyte infiltration in acute CCl_4_-induced toxicity (Baeck et al., 2012). Further insight into the precise mechanisms of CCR2/CCL2 in ALI and identification of disease settings in which modification of this axis might be of avail are required to benefit from the recent advances in the development of therapeutic strategies.

### CCL25/CCR9 pathway

Apart from the CCR2/CCL2 pathway, other chemokine networks are likely to promote monocytes/macrophages to enter the acutely inflamed liver as well. CCR9, a chemokine receptor traditionally associated with gut-homing properties of CD4^+^ T-lymphocytes in response to a CCL25 gradient, has been shown to be expressed on liver-infiltrating macrophages (Nakamoto et al., 2012). Following ConA-exposure TNF-alpha expressing CCR9^+^CD11b^+^CD11c^−^ monocytes abundantly accumulated in the liver of wild-type mice in accordance to increased hepatic *ccl25* mRNA expression. In turn, acute hepatitis was largely prevented in CCR9^−/−^ animals and severe inflammation could be restored by adoptive transfer of macrophages from *CCR9*^+/+^ mice. Concordantly, antagonizing CCL25 was able to confine ConA-induced liver damage and reduced the number of CCR9^+^ macrophages (Nakamoto et al., 2012).

### CX3CL1 (fractalkine)/CX3CR1 pathway

The CX_3_CL1 (fractalkine) receptor CX_3_CR1 is constitutively expressed on monocytes and represents the predominant chemokine receptor on the non-classical monocyte subset (Tacke and Randolph, 2006). It is not only implicated in cell migration but also controls several pleiotropic effects which are of vital importance for monocyte biology. In chronic liver injury CX_3_CL1 hepatic and serum levels are strongly regulated; CX_3_CR1 elicits liver protective functions by promoting hepatic macrophage survival and restricting adoption of a proinflammatory phenotype (Aoyama et al., 2010; Karlmark et al., 2010). Of note, CX_3_CR1-mediated macrophage survival suppresses the monocyte influx and thus virtually counteracts CCR2/CCL2 transduced promigratory effects in murine experimental injury models (Karlmark et al., 2010). Besides, CX_3_CR1 enables human CD16^+^ monocytes to migrate across inflamed sinusoidal endothelial cell layer in conjunction with vascular-adhesion-protein-1 (VAP-1; Aspinall et al., 2010). Data concerning the relevance of CX_3_CR1/CX_3_CL1 in ALI is scarce. *In situ* hybridization of *Cx_3_cl1* and *Cx3cr1* revealed increased expression of both gene transcripts in ALI (Efsen et al., 2002), although experimental liver damage was not altered in CX_3_CR1^−/−^ mice in early phases after CCl_4_-exposure (Karlmark et al., 2010). In the later time-course 72–120 h post-injury when resolution had occurred, intrahepatic CD11b^+^F4/80^+^ remained fairly stable in knockout animals instead of declining as observed in wild-type controls. In parallel, liver damage was slightly prolonged, even though differences were rather humble (Karlmark et al., 2010). Conclusively, CX_3_CL1/CX_3_CR1 interactions might spur on wound repair, though further investigations are required to underpin this theory.

## Implications of Human Monocyte and Macrophage Activity for Acute Liver Failure

Acute human liver failure is rare clinical syndrome with only 200–500 annual cases in Germany (Canbay et al., 2011). The resulting paucity of data concerning the role of human monocytes and macrophages in this syndrome significantly hampers the translation of findings from animal experimental studies into man. ALF exhibits striking parallels to the systemic inflammatory response syndrome (SIRS) and concomitant compensatory anti-inflammatory responses (CARS) observed in the evolution to septic shock, for instance (Antoniades et al., 2008; Possamai et al., 2010). Multiple organ dysfunction syndrome (MODS) represents a frequent common final path of SIRS/CARS and ALF, eventually leading to death. Therefore, the dysfunction of human monocytes/macrophages in SIRS/CARS may be regarded as a prototype for the respective processes that govern fatal outcome in ALF. The concordant acquired disturbances in the innate immune system of critically ill patients from a liver and a non-liver background have been extensively presented in a review by Antoniades et al. (2008).

### Reduced monocytic HLA-DR expression as prognostic marker in ALF

Expression of the MHC-class-II-molecule HLA-DR and *ex vivo* cytokine releasing-capacity are the most widely employed tools to study monocyte activation and function. In seminal work involving 50 patients with AALF and 20 patients with ALF due to other causes (NAALF), a dramatic reduced monocytic HLA-DR expression and declined number of total HLA-DR positive monocytes as a sign of immune paralysis could be observed in comparison to patients with stable advance chronic liver disease or healthy controls. HLA-DR levels were even more decreased on monocytes isolated from AALF patients who died or were subjected to liver transplantation in comparison AALF-subjects with transplant-free survival. The authors consecutively proposed HLA-DR expression as a marker to predict an unfavorable outcome in ALF. Simultaneous measurements of proinflammatory (IL-6, TNF-alpha, IFN-gamma) and anti-inflammatory cytokines (IL-4, IL-10) revealed highest concentrations in patients with low HLA-DR expression (Antoniades et al., 2006). The observation of markedly decreased MHC-class-II-molecule expression is highly reminiscent of the monocyte phenotype reported in numerous studies with sepsis and trauma patients and underscores the vicinity of these clinical conditions (Antoniades et al., 2008). Indeed, reduction of monocytic HLA-DR expression as marker of poor prognosis could be recapitulated in patients with acute on chronic liver injury due to superimposed bleeding and infectious complications (Wasmuth et al., 2005). In a recent study, Antoniades et al. (2012) detected depletion of circulating monocytes in AALF patients which was pronounced in individuals who died or required transplantation.

### Monocyte-released TNF-alpha and IL-10

Interestingly, *ex vivo* analysis showed a reduced capacity of monocytes to secrete TNF-alpha in response to endotoxin stimulation which was associated with poor outcome (de la Mata et al., 1990; Wigmore et al., 1998). In conjunction with reduced monocytic HLA-DR levels this suggests profound monocyte deactivation, which might contribute to immune paralysis. Immune stunning in the context of a CARS-like state is a predisposing factor to bacterial infections frequently aggravating clinical course of ALF (Antoniades et al., 2008). IL-10 is an inflammation counterbalancing cytokine that dominates the immune paralysis during CARS. Unfavorable elevated systemic IL-10 concentrations observed in ALF might thus be responsible for monocytic dysfunction since IL-10 dampens TNF-alpha release and reduces HLA-DR expression in monocytes (Koppelman et al., 1997; Possamai et al., 2010). Considering the strong capacity of CD16^−^ monocytes to secrete IL-10, it is conceivable that monocytes are simultaneously a source of and a target for IL-10. In contrast, CD16^+^ monocytes likely release TNF-alpha in conditions of ALF as well, and boost the SIRS. These oppositional roles, however, have to be scrutinized in this scenario.

### Activity of intrahepatic macrophages in human ALF

There is very sparse data aiming at the hepatic compartment in human ALF with respect to macrophages. In acetaminophen-induced liver failure locally proliferating and monocyte-derived macrophages accumulate in necrotic areas in an anti-inflammatory/regenerative niche of high IL-6, IL-10, and TGF-beta1 concentration (Antoniades et al., 2012). Increased circulating soluble CD163 (a hemoglobin scavenger receptor) levels in fulminant liver failure have been suggested to mirror intrahepatic macrophage activity and were associated with fatality (Hiraoka et al., 2005; Moller et al., 2007). Immunohistochemical studies of explanted ALF livers revealed marked expansion of CD68^+^ macrophages and augmented Fas Ligand expression by KCs, hinting at the induction of macrophage-mediated hepatocyte apoptosis through Fas–FasL interactions as a pathogenic pathway in human liver failure (Mita et al., 2005). A Japanese group proposed massively elevated serum ferritin (>5000 ng/mL) levels as surrogate marker for excessive macrophage hyperactivity, comparable to the macrophage activating syndrome (Kotoh and Takayanagi, 2010). Transarterial injection of 1000 mg methylprednisone via the *arteria hepatica propria* (a procedure named TASIT by the authors) at three consecutive days was performed in 17 patients in a single-center study. In some cases liver biopsies were obtained prior to and 1 week after the intervention showing a decline of hepatic macrophages post-treatment. In addition, survival of treated patients exceeded 70%, whereas only 24% of patients from a conservatively treated control group survived (Kotoh and Takayanagi, 2010). The authors claim a clinical benefit of this tool for selected ALI/ALF patients featuring macrophage hyperactivation as indicated by serum ferritin concentrations. However, severe limitations in the study design hamper to judge the true clinical relevance.

## Conclusion

Acute liver failure is a rare but fatal clinical condition. Despite its low prevalence it is a significant burden to public health systems. Some of the hallmark molecular events in ALI have been unraveled throughout the last decades but a considerable therapeutic breakthrough has not been accomplished so far. Indeed, new medical tools in counterbalancing the devastating inflammatory mechanisms in the acutely inflamed liver are required to prevent liver transplantation and consecutive live-long immunosuppressive therapy. Tremendous research efforts have corroborated the concept that hepatic macrophages are central in the pathogenesis of acute hepatic injury. Liver resident macrophages do not only contribute to but frequently initiate the inflammatory cascade after an acute hepatic insult. They are an important source of the prototypical proinflammatory cytokine TNF-alpha and other key mediators propagating liver injury. Liver necrosis as the macroscopic sequel to extensive hepatocyte cell death can be reverted by depletion of macrophages in many experimental models. Due to the complexity of the underlying inflammatory network, hepatic macrophages do not only compromise the integrity of liver parenchyma but also integrate sinusoidal endothelial cells and sessile as well as migrating immune cells into the detriment cascade. However, as summarized in the present review, hepatic macrophages are not exclusively deleterious. They also secrete messengers such as IL-6 or IL-10 that display local hepatoprotective properties. The ambiguous consequences of KC activation in the process of liver injury therefore might arise from a sequential release of pro- and anti-inflammatory cytokines possibly related to a classically or alternatively activated phenotype but might also be attributable to the origin of hepatic macrophages. In this light, KC-ablation even impaired liver damage in some sets of experiments. Furthermore, experimental interventions inhibiting monocyte ingress like disruption of CCR2/CCL2 and other chemokine pathways have partially deciphered the differential role of monocyte-derived macrophages in the context of ALI. Newly recruited macrophages may account for some of the dichotomous functions of hepatic macrophages that have been reported. Moreover, the nature of the underlying injury seemingly impacts macrophage function. In conclusion, although the overall contribution of monocytes and hepatic macrophages to acute hepatic injury may remain elusive, targeting macrophages exhibits a high potential for improving management of patients with ALF in the future.

## Conflict of Interest Statement

The authors declare that the research was conducted in the absence of any commercial or financial relationships that could be construed as a potential conflict of interest.

## Abbreviations

ALF, acute liver failure; ALI, acute liver injury; APAP, acetaminophen; CCl_4_, carbon tetrachloride; CCL, C-C motif chemokine ligand; CCR, C-C motif chemokine receptor; ConA, Concanavalin A; CXC, C-X-C motif chemokine; DC, dendritic cell(s); GalN, d-galactosamine; HSC, hepatic stellate cell(s); IFN, interferon; IL, interleukin; iNOs, induced nitric oxide synthetase; INR, international normalized ratio; I/R, ischemia/reperfusion; KC, Kupffer cell(s); LSEC, liver sinusoidal endothelial cell(s); LPS, lipopolysaccharide; MCP-1, monocyte-chemoattractant protein-1; NF-κB, nuclear factor kappa B; NK-cell, natural killer cell; TNF, tumor necrosis factor.
